# Determinants of Behaviour Change in a Multi-Component Telemonitoring Intervention for Community-Dwelling Older Adults

**DOI:** 10.3390/nu10081062

**Published:** 2018-08-10

**Authors:** Marije N. van Doorn-van Atten, Lisette C. P. G. M. de Groot, Jeanne H. M. de Vries, Annemien Haveman-Nies

**Affiliations:** 1 Division of Human Nutrition and Health, Wageningen University and Research, P.O. Box 17, 6700 AA Wageningen, The Netherlands; lisette.degroot@wur.nl (L.C.P.G.M.d.G.); jeanne.devries@wur.nl (J.H.M.d.V.); 2 Strategic Communication Chair, Wageningen University and Research, P.O. Box 17, 6700 AA Wageningen, The Netherlands; annemien.haveman@wur.nl

**Keywords:** older adults, diet quality, physical activity, telemonitoring, lifestyle intervention, mechanisms of impact, mediation analyses

## Abstract

Optimal diet quality and physical activity levels are essential for healthy ageing. This study evaluated the effects of a multi-component telemonitoring intervention on behavioural determinants of diet quality and physical activity in older adults, and assessed the mediating role of these determinants and two behaviour change techniques in the intervention’s effects. A non-randomised controlled design was used including 214 participants (average age 80 years) who were allocated to the intervention or control group based on municipality. The six-month intervention consisted of self-measurements of nutritional outcomes and physical activity, education, and follow-up by a nurse. The control group received regular care. Measurements took place at baseline, after 4.5 months and at the end of the study. The intervention increased self-monitoring and improved knowledge and perceived behavioural control for physical activity. Increased self-monitoring mediated the intervention’s effect on diet quality, fruit intake, and saturated fatty acids intake. Improved knowledge mediated the effect on protein intake. Concluding, this intervention led to improvements in behavioural determinants of diet quality and physical activity. The role of the hypothesised mediators was limited. Insight into these mechanisms of impact provides directions for future development of nutritional eHealth interventions for older adults, in which self-monitoring may be a promising behaviour change technique. More research is necessary into how behaviour change is established in telemonitoring interventions for older adults.

## 1. Introduction

An increasing number of older adults lives longer and healthier. An optimal nutritional status contributes to healthy ageing. Conversely, ageing poses nutritional risks as deteriorations in health, cognitive, and physical functioning, as well as changes in social circumstances, may impair nutritional status [1]. In the Netherlands, 11 to 35% of community-dwelling older adults are undernourished and diet quality of community-dwelling older adults is suboptimal [2,3]. Furthermore, awareness concerning undernutrition is low among older adults [4,5] and nutrition knowledge and attitude seem to be poorer among older adults than among younger adults [6,7,8]. Good access to appropriate nutrition care, such as meal programs, nutrition education, nutritional monitoring, counselling, and therapy, contributes to an optimal nutritional status [9]. Additionally, physical activity (PA) levels of older adults are suboptimal, with about one third of the 70–79-year-old and about half of the adults aged 80 years and over failing to meet the WHO guidelines for PA [10]. Barriers are mentioned such as health status, fear, and lack of interest [11,12], with health status also acting as a facilitator (e.g., physical benefits of PA), together with enjoyment and social support [11]. Much is expected from eHealth as a way to improve nutrition and PA behaviour [13]. Advantages of eHealth include personalisation, scalability, accessibility, and reduced costs as compared to regular face-to-face care [13].

Reviews of eHealth interventions to improve nutrition behaviour in various settings show mixed results and mostly focus on younger populations [14,15,16,17]. eHealth interventions to improve nutritional outcomes in older adults are scarce. One pilot study focussed on providing computer-tailored dietary advice to older adults, in combination with improving physical activity and meaningful social roles. This appeared to be feasible, but effectiveness has yet to be affirmed in an RCT [18]. Another eHealth study focussed on nutritional counselling for older adults at increased cardiovascular risk, but effects on dietary intake were not evaluated [19]. The scarcity of nutritional eHealth interventions for older adults and mixed results of eHealth interventions to improve nutrition behaviour in a general population call for more research.

To explore the potential of nutritional eHealth interventions for older adults, it is not only necessary to know whether interventions are effective, but also how an intervention achieves its effects [14]. Ideally, interventions rely on a theoretical framework that specifies how an intervention results in effects on behavioural determinants and behaviour through behaviour change techniques (BCT’s) [20]. Research shows that increased use of theory positively impacts effect sizes [21]. Testing a theoretical framework in order to verify the assumed relations deepens understanding of how interventions work and contributes to future intervention development. However, only a minority of nutritional eHealth studies that included a theoretical framework analysed the hypothesised mediators [14,15], and more insight is needed into what contributes to effective eHealth interventions to improve nutrition behaviour in older populations.

The PhysioDom Home Dietary Intake Monitoring (HDIM) study focused on telemonitoring of nutritional parameters and physical activity. This intervention resulted in improved compliance with the Dutch dietary guidelines for the intake of vegetables, fruit, dietary fibre, and protein, and to guidelines for PA [22]. Concerning the content of the PhysioDom HDIM intervention, the three most important BCT’s were self-monitoring, goalsetting, and feedback, reflecting an application of control theory [20,23,24]. Effectiveness of self-monitoring has been confirmed in non-eHealth studies [25], but eHealth studies including self-monitoring to promote behaviour change show less optimistic results [21,26]. It has been shown that self-monitoring is more effective in combination with other BCT’s such as goalsetting and tailored feedback [21,26]. According to the control theory, self-monitoring, goalsetting and feedback are key in behavioural self-management [24], which is relevant nowadays with the increasing focus on self-management of health and health-related behaviours [27].

All in all, we hypothesized that the intervention would result in an increased frequency of self-monitoring and goalsetting, and in improved perceived behavioural control, attitude, and knowledge, in turn improving diet quality and PA. In this article, we aimed to shed light on these hypothesised mechanisms of impact by studying changes in frequency of self-monitoring and goalsetting, by studying the effects on perceived behavioural control, attitude, and knowledge, and by studying the mediating role of self-monitoring, goalsetting, perceived behavioural control, attitude, and knowledge in the effects of PhysioDom HDIM on diet quality and PA.

## 2. Materials and Methods

### 2.1. Design

Measurements took place from April 2016 until June 2017, when the last participants finished the study. The study followed a non-randomised controlled design and had a duration of six months. Measurements took place at baseline (T0), after 4.5 months (T1), and after six months at the end of the study (T2). Telemonitoring measurements took place at the beginning of the study and three months after the start of the study, and only in the intervention group. The control group received regular care.

### 2.2. Ethics Approval and Consent to Participate

Written informed consent was obtained from all participants. The study was conducted in accordance with the Declaration of Helsinki, and all study procedures involving participants were approved by the Medical Ethical Committee of Wageningen University on the 18 February 2016, number NL53619.081.1.

### 2.3. Trial Registration

The study was registered at ClinicalTrials.gov (identifier NCT03240094), URL http://bit.ly/2zFTs3P.

### 2.4. Participants

Participants were recruited from February 2016 until September 2016 and were recruited from nine small to middle-sized municipalities in the Netherlands. Allocation of participants to the intervention or control group took place on municipality level to prevent contamination between the intervention and control group as local HCP’s implemented the intervention. Five municipalities were non-randomly allocated to the intervention group and four other municipalities were allocated to the control group. Participants were recruited via letters from the two involved care organisations, via advertisements in local newspapers and public spaces, and invitation letters via post. Inclusion criteria were being 65 years or older and receiving home care and/or living in sheltered accommodation or a service flat. Exclusion criteria were cognitive impairment (Mini Mental State Examination (MMSE) <20), having cancer, receiving terminal care, being bedridden or bound to a wheelchair, or being unable to watch television. Persons who were interested to participate were visited by a researcher to receive more information, to have questions answered, to sign the informed consent, and to be screened on the exclusion criteria.

### 2.5. Intervention

The intervention consisted of telemonitoring measurements by participants, education concerning nutrition and PA, and follow-up by a nurse. These intervention components are further described below.

#### 2.5.1. Telemonitoring Measurements

Firstly, participants performed self-measurements of body weight (weekly, with an A&D weighing scale, type UC-411PBT-C), steps (one week per month using a pedometer of A&D, type UW-101), and blood pressure (monthly or bi-monthly, only upon indication of a nurse). They also filled out questionnaires about their nutritional status, appetite, and diet quality using the Mini Nutritional Assessment Short-Form (MNA-SF) [28], Simplified Nutritional Appetite Questionnaire (SNAQ) [29], and Dutch Healthy Diet Food Frequency Questionnaire (DHD-FFQ) [30], respectively. Participants filled out these questionnaires at T0 during an interview with a researcher and three months later by means of a computer, tablet, or during a telephone interview with a researcher. Participants could view their telemonitoring results on a special television channel and could thus become aware of their nutritional status and changes in nutritional status. On this television channel, participants also received short text messages in which they were asked to write down their goals for diet quality (two times) and steps (daily, during one week per month).

#### 2.5.2. Education

Secondly, participants received computer-tailored and non-tailored information about nutrition and physical activity. The computer-tailored information consisted of advice sent per post on how to improve compliance with ten Dutch food-based dietary guidelines and the Dutch guideline for physical activity. This advice was tailored to the participant’s current compliance with the guidelines as measured by the DHD-FFQ. For example, advice concerning vegetable intake contained more accessible suggestions for participants with low compliance than for participants who were already compliant, for which suggestions were more focussed on maintaining this behaviour and diversity of vegetable intake. The non-tailored information consisted of three short television messages per week containing general information about nutrition and physical activity.

#### 2.5.3. Follow-Up

Thirdly, a total of seven nurses was available to provide follow-up on the participants’ self-measurements. On the project’s website, they checked alerts that were activated in case of undernutrition, risk of undernutrition (based on MNA score, SNAQ score, weight loss of > five percent and/or body mass index (BMI) <20 kg/m^2^), obesity (based on BMI >30 kg/m^2^), or new blood pressure measurements. Nurses planned follow-up of these alerts with help of decision trees. In case of a good nutritional status, nurses kept monitoring without taking action. In case of risk of undernutrition, nurses contacted participants via telephone or a home visit. Nurses identified causes, provided suggestions on how to improve dietary intake, and gave a leaflet with advice to reverse the risk of undernutrition [31]. In case of undernutrition or obesity, nurses discussed with participants whether referral to a dietician or general practitioner was desirable.

### 2.6. Measurements

Measurements took place during a screening visit prior to the beginning of the study and at T0, T1, and T2. In the control group, the screening visit and T0 visit coincided. Data were collected by means of structured interviews at the participant’s homes conducted by a trained researcher or research assistant. Furthermore, paper questionnaires were used to collect data.

#### 2.6.1. Baseline Characteristics

Baseline characteristics were recorded during the screening visit and at T0 and included sex, age, body weight, height, number of morbidities, education level, civil status, living situation, country of birth, cognitive functioning as measured by the Mini Mental State Examination (MMSE) [32], physical functioning as measured by the Katz-15 [33], nutritional status as measured by the Mini Nutritional Assessment (MNA) [34], desire to lose weight, and type of received care. Body weight was measured without shoes and heavy clothes (scale of type UC-411PBT-C, A&D).

#### 2.6.2. Frequency of Self-Monitoring and Goalsetting

Frequency of self-monitoring and goalsetting was measured at T0, T1, and T2 using a paper questionnaire with items derived from literature (Table 1). Self-monitoring was measured using four statements that were combined to form one scale for self-monitoring [35]. Goalsetting was measured using three statements that were combined to form one scale for goalsetting [36].

#### 2.6.3. Behavioural Determinants

Behavioural determinants were measured at T0, T1, and T2 using a paper questionnaire with items derived from literature (Table 1). Perceived behavioural control (PBC) was measured using two items for self-efficacy and two items for controllability for both physical activity (PA) and healthy eating (HE) behaviour [37]. These items were combined into two scales for PA and HE. Attitude concerning HE and PA were each measured by six semantic differential items [38]. Items were combined to form scales of attitude concerning PA and HE. Crohnbach’s alpha’s for the abovementioned questionnaire items ranged from 0.67 to 0.80. Knowledge was measured using 11 statements concerning a healthy diet and physical activity that were answered with ‘true’, ‘false’, or ‘I don’t know’. A knowledge score (0–11) was generated based on the number of correct answers.

#### 2.6.4. Compliance with Dutch Dietary Guidelines and Guidelines for Physical Activity

Compliance with Dutch dietary guidelines and guidelines for physical activity were evaluated using the DHD-FFQ, which was administered during a structured interview at T0 and T2. The DHD-FFQ contains 29 items that evaluate compliance with Dutch dietary guidelines and compliance with PA guidelines [30,39]. Additionally, for this study, compliance with guidelines for the intake of protein and vitamin D was evaluated as the DHD-FFQ contains questions on all relevant protein and vitamin D sources consumed by a Dutch elderly population [3,40]. This resulted in sub scores for compliance with guidelines for the intake of fruit (≥200 g), vegetables (≥150–200 g), dietary fibre (≥14 g/4.2 MJ), fish (two times per week, from which at least one time fatty fish), saturated fatty acids (<10 en%), trans fatty acids (<1 en%), salt (<6 g), alcohol (≤2 glasses for men, ≤1 glass for women), protein (≥70 grams for men, ≥55 grams for women), vitamin D (≥20 mcg), and physical activity (moderate physical activity for at least 30 min a day on at least five days a week). These scores ranged from 0–10, with higher scores indicating better compliance. A total score for diet quality was constructed by summing scores for vegetables, fruit, dietary fibre, fish, alcohol, saturated fatty acids, trans-fatty acids, and sodium. More information can be found elsewhere [30].

### 2.7. Statistics

The sample size was based on the primary outcome of the main study: nutritional status [34]. Aiming to detect a difference in MNA change of three, assuming a standard deviation of 6.1 [41], and taking into account a two-sided significance level of 0.05 and power of 80%, a sample size of 65 participants per group was required based on the formula $2\times \frac{\left[{\left(Z\alpha /2+Z\beta \right)}^{2}\times \sigma^{2}\right]}{\delta^{2}}$. We expected a drop-out rate of 30% at maximum, therefore we needed a sample size of at least 93 participants in each group.

**Figure 1:**
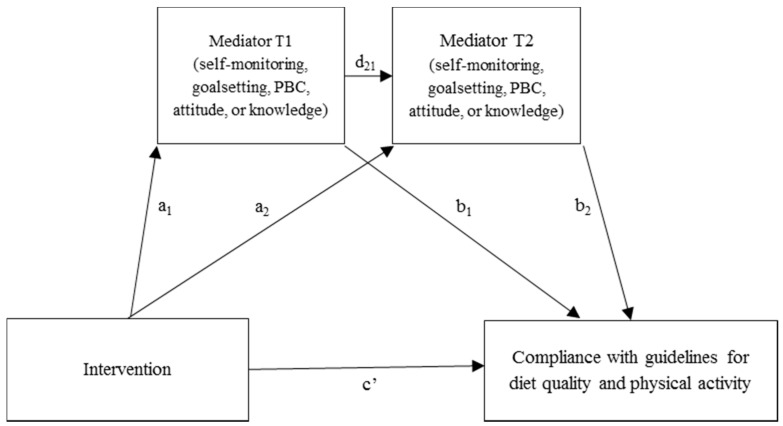
Hypothesised mediation pathways in the PhysioDom Home Dietary Intake Monitoring (HDIM) intervention. One model for each outcome and mediator.

Data were analysed using SPSS Statistics for Windows version 22 (IBM Corp., Armonk, NY, USA). Statistical analyses were carried out according to the intention-to-treat principle. Firstly, baseline characteristics of the intervention and control group were described using means (± standard deviations) or percentages. Differences between the groups were tested using independent *t*-tests, Mann-Whitney *U* tests in case of non-normality, or chi-square tests. Secondly, changes in self-monitoring and goalsetting and intervention effects on behavioural determinants were assessed using linear mixed models. Therefore, we first specified a model as large as possible including all main effects, possible interactions, and an unstructured covariance matrix. We then simplified the random part of the model by testing whether simpler covariance structures were allowed using the (REML) LR test, until a model was obtained that was as parsimonious as possible. Consequently, we simplified the fixed part of the model by including dummies for time points T1 and T2, treatment, the interaction terms of the time point dummies and treatment, age, sex, and also other covariates (e.g., BMI, education level, living situation, MNA, functional status, MMSE, municipality, diet, informal care) if they considerably (e.g., >10%) influenced the effect estimates. Thirdly, we performed mediation analyses to evaluate whether the effects of PhysioDom HDIM were mediated as hypothesised (Figure 1). The following outcomes were selected for mediation analyses as these were positively affected by the PhysioDom HDIM intervention: compliance with the Dutch dietary guidelines for the intake of fruit, vegetables, fibre, and protein, and compliance with Dutch guidelines for PA [22]. As mediation can also exist in absence of a significant intervention effect on the study’s outcomes [42], also other components of the DHD-FFQ (alcohol, salt, saturated fatty acids, fish, and for this study vitamin D) and the total DHD-FFQ score were included as outcomes in the mediation analyses. To capture the longitudinal nature of the data, we used a multiple serial mediation model for each outcome and each hypothesised mediator in which the mediator at T1 and T2 was modelled in sequence (Figure 1). We regarded a parallel multiple mediator model less appropriate as the condition that no mediator causally influences another would probably not be fulfilled [42]. Using the PROCESS macro for SPSS version 2.16.3 we assessed whether indirect effects of the intervention on the selected outcomes through the hypothesised mediators were statistically significant [42]. Standard errors and confidence intervals of indirect effects were calculated using bootstrapping (10,000 samples). The analyses for diet quality and physical activity were adjusted for age, sex, and baseline values of the mediator.

## 3. Results

### 3.1. Baseline Characteristics

**Figure 2:**
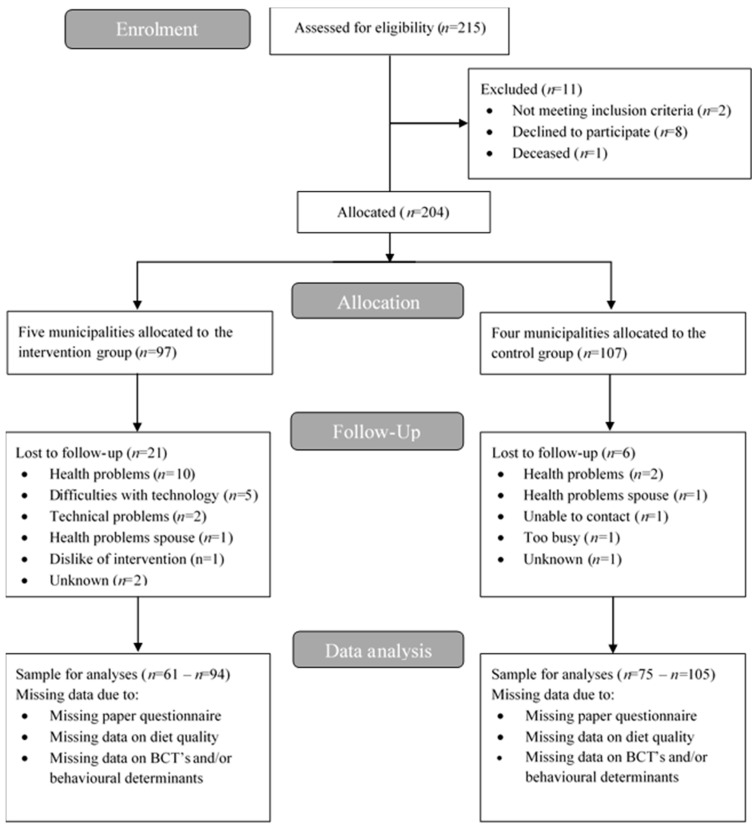
Flow diagram of participants of the PhysioDom HDIM study.

In total, 215 persons were screened, from which 97 were allocated to the intervention group and 107 to the control group. During the study, 21 intervention group participants and six control group participants were lost to follow-up. A flow chart with reasons for loss to follow-up can be found in Figure 2. Table 2 shows the baseline characteristics of the intervention and control group. Participants in the intervention group were slightly younger and had a higher BMI than participants in the control group. Participants in the intervention group lived less often alone and received more often informal care than control group participants.

### 3.2. Changes in Self-Monitoring and Goalsetting and Effects on Behavioural Determinants

Table 3 shows changes in self-monitoring and goalsetting and shows the effects of the intervention on behavioural determinants. At baseline, there were no significant differences between the intervention and control group. During the intervention, several significant changes were observed. Firstly, the intervention group significantly increased scores for self-monitoring at T1 and T2, compared to the control group (T1: β = 0.49, 95% CI 0.19, 0.80; T2: β = 0.50, 95% CI 0.20, 0.80). Secondly, intervention participants perceived an increased behavioural control for physical activity at T2 compared to the control group (β = 0.26, 95% CI 0.08, 0.45). Thirdly, participants in the intervention group improved their knowledge at T1 and T2 compared to the control group, with the improvement at T2 being significant (β = 0.51, 95% CI 0.04, 0.99).

### 3.3. Effect Mediation

Four significant mediation pathways were found. Firstly, the effect of the intervention on compliance with the guidelines for the intake of fruit was mediated by increased self-monitoring behaviour at T1. Secondly, the effect of the intervention on compliance with the guidelines for the intake of protein was mediated by improvements in knowledge at T1 and T2 (Table 4). Thirdly, even though a significant effect of the intervention on the total DHD-FFQ score was lacking, we found significant mediation by self-monitoring at T1 (Appendix A). Likewise, increased self-monitoring mediated the intervention’s effect on compliance with guidelines for the intake of saturated fat (Appendix A).

## 4. Discussion

This study aimed to evaluate the effects of a multi-component telemonitoring intervention on behavioural determinants of nutrition and physical activity behaviour in older adults, and to evaluate the role of mediators in the effects on behaviour. The intervention resulted in improvements in self-monitoring, perceived behavioural control for physical activity, and knowledge. Furthermore, self-monitoring mediated the effect of the intervention on total diet quality score and compliance with the guidelines for the intake of fruit and saturated fatty acids. Knowledge mediated the effect of the intervention on compliance with the guidelines for the intake of protein.

Intervention group participants increased their self-monitoring, which mediated the effect of the intervention on total DHD-FFQ score, and the intake of fruit and saturated fatty acids. Scores for self-monitoring improved from 2.9 at T0 to 3.5 at T1 and 3.3 at T2, meaning that the frequency of self-monitoring increased from on average a few times per month to somewhere between a few times per month and weekly, suggesting that this rather small change is already sufficient to partly mediate the intervention’s effect. Self-monitoring of diet, physical activity, and weight has mainly been used in weight loss programs with more frequent self-monitoring resulting in more weight loss as compared to less frequent self-monitoring [43]. Another study focussing on the effects of self-monitoring by means of mobile devices showed positive outcomes on dietary intake [44]. In an intervention study, the effect of daily tailored messaging on weight loss was mediated by self-monitoring of diet and physical activity [45]. In our study, self-monitoring mediated the effect of the intervention on total diet quality score and the intake of fruit and saturated fatty acids, but not the effect on other diet quality components. A possible explanation is that self-monitoring of diet was not very intensive as participants filled out the DHD-FFQ twice during the six-month intervention, as opposed to more frequent dietary self-monitoring encountered in other studies [44]. Apparently, also other mechanisms besides self-monitoring have caused the intervention to result in positive changes in diet quality and physical activity. Increasing the frequency of self-monitoring of dietary intake may strengthen the effect of this intervention.

The intervention resulted in increased perceived behavioural control for PA, but this did not mediate the intervention’s effect on physical activity. This seems contradictory to the theory of planned behaviour that poses that PBC precedes behavioural intention and behaviour [46]. Furthermore, PBC is seen as a predictor of the translation of intention into behaviour [47,48]. With regard to older adults, PBC is considered as a consistent predictor of physical activity initiation and maintenance [49]. In contrast, an intervention study aiming to improve physical activity among participants with increased risk of type 2 diabetes shows that PBC did not predict physical activity or change in physical activity [50]. The authors argue that the TPB may be less accurate in explaining behaviour among clinical samples than among the often-used student samples, which could also explain the lack of mediation by PBC in this study [50]. All in all, other mechanisms besides the ones that we have measured may have been important in increasing physical activity levels of our study population, for example, awareness, enjoyment, or action planning [49].

The intervention had a positive effect on knowledge, and this mediated the intervention’s effect on compliance with the guidelines for the intake of protein, but not the effects on compliance with other dietary guidelines. Knowledge score improved from 7.3 at T0 to 8.3 at T2, meaning that intervention group participants were able to answer one more knowledge statement correctly, from the eleven statements in total. This rather small effect size may explain why the intervention’s effect was only limitedly mediated via knowledge. Nutrition education intervention studies among older adults have shown positive effects on knowledge [51,52,53], although in the study by Racine et al, this was not associated with better adherence to the DASH diet [51,52]. A review examining the relationship between nutrition knowledge and dietary intake showed positive but weak correlations [54]. The general idea is that nutritional knowledge is necessary but not sufficient for changing dietary habits, and that the association of knowledge with diet quality is complex and influenced by many other demographic and environmental factors [54]. Furthermore, the knowledge questionnaire used in this study assessed declarative knowledge, while procedural knowledge of nutrition (e.g., knowing how to read food labels or how to cook a healthy meal) might be more relevant for making healthy food choices [55]. Nevertheless, improved knowledge did mediate the intervention’s effect on the compliance with dietary guidelines for protein intake. This is a relevant finding, as sufficient protein intake in older adults is necessary to counteract age-related loss of muscle mass [56]. Furthermore, older adults seem unaware of the importance of sufficient intake of protein [4]. This study suggests that increasing nutritional knowledge might be an effective and relatively easy way to improve protein intake in older adults.

The intervention did not result in significant changes in goalsetting, attitude, and perceived behavioural control for healthy eating. Several possible explanations could be given. The emphasis of the intervention was on self-monitoring of nutritional outcomes and PA. Participants received training and instructions to do these self-measurements and were reminded via a paper calendar and television messages, resulting in increased self-monitoring behaviour. Participants were also prompted to set goals for diet quality and PA, but only via the intervention manual and via television messages, which were not always read. This could explain the lack of significant effects on goal-setting. Secondly, attitude and perceived behavioural control for healthy eating were also targeted through television messages. Again, too little messages might have been read to have an impact on these behavioural determinants. Furthermore, television messages to target PBC for healthy eating were mainly focussed on the individual. However, PBC might also be affected by characteristics that are not easily targeted, such as impaired physical functioning, limited mobility, limited cooking skills, or more environmental determinants such as distance to a supermarket. All in all, to target goalsetting, attitude, and perceived behavioural control for healthy eating, a higher intervention dose might be necessary to result in change.

To our knowledge, this is the first study that aimed to unravel mechanisms of impact of an intervention that focussed on improving nutritional status in community-dwelling elderly through eHealth. Strengths of this study include the use of a theoretical framework and validated constructs to measure behavioural determinants. This study also has limitations that may have contributed to the limited significant findings from the mediation analyses. The population for analysis was smaller for the mediation analyses than for the mixed model analyses, as the method used for mediation analyses is less flexible concerning missing data. This could have resulted in a loss of power or have obscured mediation pathways. Secondly, using self-report measures of diet and physical activity instead of objective measures of behaviour may have led to weaker associations with the proposed behavioural determinants [57]. Furthermore, older adults might be less good in filling out TPB questionnaires than younger adults [50]. Thirdly, it is uncertain whether BCT’s which have been proven successful in younger populations can be applied in older populations as well [58]. It may well be that some BCT’s may be too complex for older adults with impaired physical functioning or in another way do not appeal to older adults, potentially explaining the limited results from the mediation analyses. Finally, it is uncertain whether effects on behavioural determinants and behaviour will sustain. Participants could keep the weighing scale and pedometer to keep track of their weight and daily steps. Employing BCT’s enhances the chance that participants maintain their behaviour [13]. On the other hand, this study mainly focussed on individual determinants of health behaviour, while it is suggested that organisational and societal determinants are also important for achieving sustained change [59]. More research is necessary to assess the long-term effectiveness of nutritional eHealth interventions, and what exactly contributes to long-term impact. 

Finally, this study showed that a multi-component telemonitoring intervention for community-dwelling older adults resulted in increased self-monitoring behaviour, and improved perceived behavioural control for physical activity and knowledge. The intervention’s effect on total diet quality score, fruit intake, and saturated fatty acids intake was mediated by self-monitoring and the effect on protein intake was mediated by knowledge. Apparently, other unknown mediators have also played an important role in achieving the intervention’s results on diet quality and physical activity. This calls for more research into which behaviour change techniques are effective in improving nutritional outcomes in older adults.

## Figures and Tables

**Table 1:** Items to measure self-monitoring, goalsetting, perceived behavioural control, and attitude in the PhysioDom HDIM study.

Construct	Questionnaire Items	Answering Options (1–5)	Crohnbach’s Alpha T0
Self-monitoring	How often in the past month have you kept track in your head of the amount of food you have eaten? How often in the past month have you kept track in your head of the types of foods you have eaten during the course of the day? How often in the past month have you kept track in your head of how physically active you have been during a week? How often in the past month you have weighed yourself?	Never/a single time/a couple of times/every week/everyday	0.77
Goalsetting	How often in the past month did you set goals related to your weight? How often in the past month did you set goals related to your eating habits? How often in the past month did you set goals related to how much you exercise?	Never/a single time/a couple of times/every week/everyday	0.67
Perceived behavioural control healthy eating	I am confident that I can eat healthy in the coming month if I want to. Whether I eat healthy in the coming month is entirely up to me. Healthy eating in the coming month is for me... How much control do you have over healthy eating in the coming month?	Totally disagree–totally agree Totally disagree–totally agree Difficult–easy No control–complete control	0.70
Perceived behavioural control physical activity	I am confident that I can be sufficiently physically active in the coming month if I want to. Whether I am sufficiently physically active in the coming month is entirely up to me. Sufficient physical activity in the coming month is for me... How much control do you have over being sufficiently physically active in the coming month?	Totally disagree–totally agree Totally disagree–totally agree Difficult–easy No control–complete control	0.71
Attitude healthy eating	Healthy eating in the coming month is for me...	foolish–wise pleasant–unpleasant bad–good harmful–helpful unnecessary–necessary unenjoyable–enjoyable	0.70
Attitude physical activity	Physical activity in the coming month is for me...	foolish–wise pleasant–unpleasant bad–good harmful–helpful unnecessary–necessary boring–interesting	0.80

**Table 2:** Baseline characteristics of participants of the PhysioDom HDIM study.

	Intervention Group (*n* = 97)		Control Group (*n* = 107)		*p*-Value ^a^
	Mean	*SD*	Mean	*SD*	
Age (years)	78.4	7.2	81.0	7.9	0.02
BMI (kg/m^2^)	29.2	4.5	27.7	5.4	0.04
Number of diagnoses	1.5	1.5	1.3	1.3	0.26
MMSE score	28.6	1.5	25.8	1.9	0.69
Katz-15 score	Mdn	IQR	Mdn	IQR	
	1.0	0–4	1.0	0–3	0.69
	Percentage		Percentage		
Sex (male)	35		23.4		0.09
Education level ^b^					0.08
Low	17.5		10.3		
Moderate	55.7		49.5		
High	26.8		40.2		
Civil status					0.11
Married	42.3		27.1		
Single	7.2		13.1		
Divorced	7.2		10.3		
Widowed	43.3		49.5		
Living alone	55.7		74.8		0.004
Born in the Netherlands	96.9		90.7		0.07
Desire to lose weight	52.7		39.4		0.07
Nutritional status					0.45
Normal nutritional status	79.2		83.8		
At risk of undernutrition	19.8		16.2		
Undernourished	1.0		0.0		
Type of care					
Domestic care	78.4		80.4		0.72
Personal care	32.0		29.9		0.75
Nursing care	9.3		2.8		0.05
Individual support	3.1		0.9		0.27
Informal care	32.0		11.2		<0.001

*SD*, Standard Deviation; BMI, Body Mass Index; MMSE, Mini-Mental State Examination. ^a^ Independent *t*-test, Mann-Whitney test, or chi-square test. ^b^ Low education level: primary school or less; intermediate level of education: secondary professional education or vocational school; High education level: higher vocational education, university.

**Table 3:** Changes in self-monitoring and goalsetting and effects of the PhysioDom HDIM intervention on knowledge, perceived behavioural control, and attitude.

	Intervention Group						Control Group						Linear Mixed Models		
	T0		T1		T2		T0		T1		T2		β T1 (95% CI)	β T2 (95% CI)	*N*
	Mean	*SD*	Mean	*SD*	Mean	*SD*	Mean	*SD*	Mean	*SD*	Mean	*SD*			
Self-monitoring	2.9	1.2	3.5	0.9	3.3	1.1	3.1	1.2	3.1	1.2	3.0	1.3	0.49 (0.19, 0.80) **	0.50 (0.20, 0.80) **	199
Goalsetting	2.7	1.2	3.0	1.1	2.8	1.2	3.0	1.1	3.0	1.3	2.9	1.2	0.25 (−0.05, 0.55)	0.19 (−0.10, 0.48)	199
Knowledge ^a^	7.3	2.1	8.2	1.9	8.3	1.8	7.5	2.0	7.5	2.1	7.6	2.2	0.51 (−0.09, 1.12)	0.51 (0.04, 0.99) *	198
PBC HE ^b^	4.1	0.7	4.2	0.7	4.2	0.6	4.3	0.6	4.1	0.8	4.3	0.7	0.16 (−0.02, 0.33)	0.08 (−0.09, 0.25)	188
PBC PA	3.7	0.8	3.8	0.9	3.9	0.9	4.0	0.9	3.8	1.0	3.9	0.9	0.19 (−0.03, 0.41)	0.26 (0.08, 0.45) **	199
Attitude HE ^c^	4.7	0.5	4.7	0.5	4.7	0.5	4.6	0.5	4.6	0.5	4.6	0.6	−0.01 (−0.18, 0.16)	0.00 (−0.17, 0.17)	188
Attitude PA ^d^	4.5	0.6	4.4	0.8	4.5	0.7	4.5	0.7	4.4	0.8	4.5	0.7	−0.04 (−0.29, 0.22)	−0.05 (−0.26, 0.15)	190

SD, standard deviation; CI, confidence interval; PBC: perceived behavioural control; HE: healthy eating; PA: physical activity. All results are adjusted for age and sex. ^a^ Adjusted for age, sex, and Mini Mental State Examination score. ^b^ Adjusted for age, sex, BMI, living situation, nutritional status, and physical functioning. ^c^ Adjusted for age, sex, BMI, physical functioning and cognitive functioning. ^d^ Adjusted for age, sex, BMI, physical functioning. * *p *< 0.05; ** *p *< 0.01.

**Table 4:** Mediation of the intervention’s effect on diet quality and physical activity.

	Indirect Effect 1 ^a,b^ (a_1_ × b_1_)		Indirect Effect 2 ^a,c^ (a_1_ × d_21_ × b_2_)		Indirect Effect 3 ^a,d^ (a_2_ × b_2_)		
	β (SE)	95% CI	β (SE)	(95% CI)	β (SE)	(95% CI)	*N*
T0–T2 Fruit							
Self-monitoring	0.16 (0.10)	0.02, 0.45	0.02 (0.04)	−0.04, 0.13	0.04 (0.08)	−0.11, 0.24	141
Goalsetting	0.03 (0.08)	−0.08, 0.27	−0.00 (0.03)	−0.09, 0.03	−0.01 (0.04)	−0.15, 0.03	140
Knowledge	0.17 (0.14)	−0.01, 0.57	−0.02 (0.05)	−0.15, 0.05	−0.02 (0.06)	−0.21, 0.04	139
PBC HE	0.01 (0.05)	−0.05, 0.18	0.02 (0.03)	−0.01, 0.13	−0.02 (0.05)	−0.20, 0.04	136
Attitude HE	−0.00 (0.04)	−0.09, 0.07	0.00 (0.01)	−0.01, 0.03	−0.00 (0.05)	−0.12, 0.07	137
T0–T2 Vegetables							
Self–monitoring	−0.07 (0.11)	−0.11, 0.35	−0.06 (0.05)	−0.22, 0.01	−0.12 (0.11)	−0.39, 0.04	141
Goalsetting	0.01 (0.06)	−0.05, 0.22	−0.00 (0.03)	−0.07, 0.05	−0.00 (0.05)	−0.12, 0.08	140
Knowledge	−0.07 (0.09)	−0.32, 0.06	−0.01 (0.05)	−0.11, 0.08	−0.01 (0.06)	−0.18, 0.08	139
PBC HE	−0.01 (0.07)	−0.25, 0.06	−0.01 (0.03)	−0.13, 0.02	0.02 (0.05)	−0.04, 0.21	136
Attitude HE	−0.01 (0.07)	−0.23, 0.07	0.00 (0.01)	−0.01, 0.04	−0.00 (0.05)	−0.13, 0.08	137
T0–T2 Dietary fibre							
Self-monitoring	−0.07 (0.07)	−0.27, 0.03	0.03 (0.04)	−0.02. 0.14	0.07 (0.07)	−0.05, 0.26	141
Goalsetting	−0.00 (0.03)	−0.08, 0.05	0.01 (0.02)	−0.02, 0.09	0.02 (0.04)	−0.02, 0.15	140
Knowledge	0.02 (0.06)	−0.06, 0.22	0.01 (0.03)	−0.04, 0.10	0.1 (0.04)	−0.04, 0.14	139
PBC HE	0.05 (0.05)	−0.03, 0.20	−0.01 (0.02)	−0.08, 0.01	0.01 (0.03)	−0.02, 0.13	136
Attitude HE	0.00 (0.03)	−0.05, 0.06	0.00 (0.01)	−0.00, 0.03	−0.00 (0.02)	−0.09, 0.03	137
T0–T2 Protein							
Self-monitoring	−0.25 (0.19)	−0.76, 0.00	0.00 (0.08)	−0.16, 0.18	0.00 (0.17)	−0.37, 0.34	141
Goalsetting	−0.04 (0.12)	−0.42, 0.12	0.01 (0.06)	−0.05, 0.23	0.04 (0.09)	−0.05, 0.37	140
Knowledge	−0.07 (0.12)	−0.44, 0.07	0.12 (0.09)	0.006, 0.41	0.12 (0.13)	−0.04, 0.52	139
PBC HE	0.06 (0.09)	−0.04, 0.40	−0.02 (0.04)	−0.17, 0.02	0.02 (0.07)	−0.05, 0.28	136
Attitude HE	0.01 (0.07)	−0.08, 0.23	0.00 (0.01)	−0.01, 0.06	−0.01 (0.06)	−0.20, 0.08	137
T0–T2 Physical activity							
Self-monitoring	−0.02 (0.16)	−0.40, 0.29	−0.04 (0.08)	−0.27, 0.07	−0.10 (0.17)	−0.52, 0.18	141
Goalsetting	−0.02 (0.08)	−0.32, 0.07	−0.00 (0.05)	−0.51, 0.07	−0.01 (0.08)	−0.25, 0.10	140
Knowledge	−0.10 (0.13)	−0.49, 0.07	−0.04 (0.07)	−0.24, 0.05	−0.04 (0.09)	−0.35, 0.05	139
PBC PA	−0.04 (0.10)	−0.31, 0.10	0.07 (0.08)	−0.01, 0.34	0.16 (0.13)	−0.02, 0.51	137
Attitude PA	−0.003 (0.05)	−0.14, 0.07	0.001 (0.02)	−0.04, 0.06	0.008 (0.05)	−0.06, 0.17	133

SE: standard error; CI: confidence interval; PBC: perceived behavioural control; HE: healthy eating, PA: physical activity. All results were adjusted for age and sex. ^a^ Standard errors and confidence intervals for indirect effects were calculated with bootstrapping (10,000 samples). ^b^ Indirect effect of the intervention on the outcome Y through the mediator at T1. ^c^ Indirect effect of the intervention on the outcome Y through the mediators at T1 and T2 in serial. ^d^ Indirect effect of the intervention on the outcome Y through the mediator at T2.

## Appendix A

**Table A1:** Mediation of the intervention’s effect on total diet quality score and other diet quality components.

	Indirect Effect 1 ^a,b^ (a_1_ × b_1_)		Indirect effect 2 ^a,c^ (a_1_ × d_21_ × b_2_)		Indirect Effect 3 ^a,d^ (a_2_ × b_2_)		
	β (SE)	95% CI	β (SE)	(95% CI)	β (SE)	(95% CI)	*N*
T0–T2 Total DHD-FFQ score							
Self-monitoring	0.79 (0.50)	0.09, 2.11	0.07 (0.21)	−0.27, 0.65	0.16 (0.47)	−0.61, 1.35	141
Goalsetting	0.07 (0.30)	−0.24, 1.06	0.04 (0.16)	−0.15, 0.65	0.11 (0.26)	−0.15, 1.06	140
Knowledge	0.23 (0.37)	−0.28, 1.32	−0.17 (0.19)	−0.74, 0.07	−0.18 (0.27)	−1.10, 0.11	139
PBC HE	0.05 (0.23)	−0.28, 0.76	0.01 (0.11)	−0.20, 0.27	−0.01 (0.16)	−0.44, 0.28	136
Attitude HE	−0.03 (0.16)	−0.55, 0.19	−0.01 (0.06)	−0.19, 0.05	0.05 (0.24)	−0.30, 0.80	137
T0–T2 Fish							
Self-monitoring	−0.07 (0.11)	−0.36, 0.10	0.01 (0.06)	−0.09, 0.14	0.02 (0.12)	−0.20, 0.30	141
Goalsetting	−0.01 (0.07)	−0.23, 0.07	−0.00 (0.03)	−0.08, 0.06	−0.00 (0.05)	−0.14, 0.09	140
Knowledge	0.09 (0.10)	−0.04, 0.38	−0.02 (0.06)	−0.17, 0.08	−0.02 (0.07)	−0.24, 0.08	139
PBC HE	0.05 (0.09)	−0.04, 0.35	−0.02 (0.04)	−0.18, 0.02	0.02 (0.06)	−0.04, 0.22	136
Attitude HE	0.00 (0.04)	−0.08, 0.09	−0.00 (0.01)	−0.04, 0.01	0.01 (0.05)	−0.06, 0.17	137
T0–T2 Saturated fatty acids							
Self-monitoring	0.31 (0.21)	0.02, 0.86	0.06 (0.09)	−0.05, 0.32	0.14 (0.18)	−0.14, 0.59	141
Goalsetting	0.05 (0.16)	−0.17, 0.51	−0.00 (0.06)	−0.18, 0.10	−0.01 (0.10)	−0.30, 0.15	140
Knowledge	0.03 (0.15)	−0.22, 0.40	−0.03 (0.08)	−0.24, 0.09	−0.03 (0.10)	−0.35, 0.09	139
PBC HE	−0.01 (0.10)	−0.29, 0.16	0.04 (0.06)	−0.03, 0.27	−0.04 (0.10)	−0.39, 0.08	136
Attitude HE	−0.03 (0.12)	−0.46, 0.12	−0.01 (0.03)	−0.12, 0.02	0.03 (0.12)	−0.15, 0.37	137
T0–T2 Salt							
Self-monitoring	0.05 (0.13)	−0.17, 0.37	0.02 (0.07)	−0.10, 0.20	0.04 (0.15)	−0.23, 0.40	141
Goalsetting	−0.00 (0.07)	−0.18, 0.12	0.01 (0.05)	−0.04, 0.19	0.03 (0.08)	−0.05, 0.34	140
Knowledge	0.00 (0.11)	−0.21, 0.27	−0.08 (0.07)	−0.32, 0.01	−0.08 (0.10)	−0.42, 0.03	139
PBC HE	−0.06 (0.09)	−0.37, 0.04	0.02 (0.04)	−0.02, 0.15	−0.02 (0.06)	−0.23, 0.04	136
Attitude HE	0.01 (0.05)	−0.05, 0.16	−0.00 (0.02)	−0.05, 0.02	0.01 (0.07)	−0.10, 0.20	137
T0–T2 Alcohol							
Self-monitoring	0.04 (0.04)	−0.02, 0.17	0.01 (0.04)	−.0.05, 0.11	0.02 (0.08)	−0.13, 0.20	141
Goalsetting	−0.00 (0.03)	−0.10, 0.05	0.00 (0.02)	−0.01, 0.06	0.01 (0.02)	−0.01, 0.09	140
Knowledge	−0.00 (0.03)	−0.08, 0.05	0.01 (0.03)	−0.03, 0.11	0.01 (0.04)	−0.03, 0.15	139
PBC HE	0.01 (0.02)	−0.01, 0.07	0.01 (0.02)	−0.01, 0.11	−0.01 (0.04)	−0.18, 0.03	136
Attitude HE	0.00 (0.03)	−0.02, 0.10	−0.00 (0.02)	−0.08, 0.01	0.01 (0.08)	−0.07, 0.31	137
T0–T2 Vitamin D							
Self-monitoring	0.00 (0.04)	−0.07, 0.08	−0.01 (0.03)	−0.06, 0.06	−0.01 (0.06)	−0.14, 0.11	141
Goalsetting	−0.00 (0.02)	−0.07, 0.03	−0.00 (0.01)	−0.05, 0.01	−0.01 (0.02)	−0.09, 0.01	140
Knowledge	0.01 (0.03)	−0.05, 0.08	0.03 (0.04)	−0.01, 0.16	0.04 (0.05)	−0.01, 0.24	139
PBC HE	0.02 (0.0.3)	−0.01, 0.12	−0.02 (0.03)	−0.12, 0.01	0.02 (0.05)	−0.03, 0.17	136
Attitude HE	0.00 (0.02)	−0.03, 0.08	−0.00 (0.00)	−0.02, 0.01	0.00 (0.02)	−0.03, 0.06	137

SE: standard error; CI: confidence interval; PBC: perceived behavioural control; HE: healthy eating, PA: physical activity. All results were adjusted for age and sex. ^a^ Standard errors and confidence intervals for indirect effects were calculated with bootstrapping (10,000 samples). ^b^ Indirect effect of the intervention on the outcome Y through the mediator at T1. ^c^ Indirect effect of the intervention on the outcome Y through the mediators at T1 and T2 in serial. ^d^ Indirect effect of the intervention on the outcome Y through the mediator at T2.
